# Does Dorsal Preservation Rhinoplasty Preserve Nasal Airway Function? A Systematic Review

**DOI:** 10.1017/S0022215125104052

**Published:** 2026-02

**Authors:** Niall James McInerney, Jack Eustace, Mohamed Amin

**Affiliations:** 1 Department of Otolaryngology, Mater Misericordiae University Hospital, Dublin, Ireland; 2 Royal College of Surgeons in Ireland, Dublin, Ireland; 3 School of Medicine, University College Dublin, Dublin, Ireland

**Keywords:** dorsal preservation rhinoplasty, septorhinoplasty

## Abstract

**Objectives:**

This study aimed to systematically review the evidence on functional outcomes following dorsal preservation rhinoplasty, with a focus on nasal obstruction.

**Methods:**

A systematic review was conducted according to Preferred Reporting Items for Systematic reviews and Meta-Analyses guidelines. PubMed, Embase, Scopus and Cochrane databases were searched up to March 2025. Studies reporting nasal airway outcomes following dorsal preservation rhinoplasty using subjective or objective measures were included.

**Results:**

Six studies comprising 662 patients were included. Patient-reported outcomes (Nasal Obstruction Symptom Evaluation, Standardized Cosmesis and Health Nasal Outcomes Survey and Visual Analogue Scale) consistently demonstrated significant post-operative improvement. Objective measures (acoustic rhinometry, rhinomanometry and cone-beam computed tomography) showed maintained or improved airway dimensions. dorsal preservation rhinoplasty was functionally equivalent to traditional structural techniques, with low complication (<5 per cent) and revision rates (<2 per cent).

**Conclusion:**

Dorsal preservation rhinoplasty maintains or improves nasal airway function while preserving structural integrity and reducing the need for grafting. It is a safe and effective alternative to traditional dorsal reduction techniques.

## Introduction

Rhinoplasty is one of the most common facial plastic surgery procedures, balancing functional and aesthetic goals.^1^ In traditional dorsal hump reduction, the nasal dorsum is deconstructed reducing the hump with resection.^2^^,^^3^ It often requires reconstruction of the mid-vault using spreader grafts, flaps or sutures, which can also improve the nasal airway.^4^ However, anatomical disruption carries risks: mid-vault collapse, nasal valve compromise, irregular dorsal aesthetic lines and the need for grafts or revision procedures.^5^^–^^7^ Functional sequelae, such as breathing impairment, can be significant. Thus, in recent decades, surgeons have sought techniques that preserve nasal structure while achieving aesthetic goals.

Dorsal preservation rhinoplasty (DPR) offers an alternative option. DPR techniques (e.g., push-down or let-down) lower the entire dorsum, which maintains dorsal continuity, preserves the keystone area and ensures mid-vault stability. First hinted at in early 20th-century techniques, DPR was revitalised by Saban in modern rhinoplasty literature and refined by various authors to reduce reliance on grafting and mid-vault reconstruction.^8^^–^^10^ Advocates argue that by preserving natural anatomy, patients fare better functionally, with fewer complications and more natural post-operative dorsal lines.^11^

Despite growing popularity, functional outcomes after DPR are not yet fully understood. Some critics caution that altering dorsal height as a unit could narrow the airway or cause internal valve collapse if not properly supported. Objective measures (acoustic rhinometry, rhinomanometry and computed tomography [CT]) and subjective metrics (NOSE [Nasal Obstruction Symptom Evaluation], SCHNOS [Standardized Cosmesis and Health Nasal Outcomes Survey], SNOT-22 [22-item Sino-Nasal Outcome Test] and Visual Analogue Scales [VAS]) provide tools to assess post-operative airway patency and patient satisfaction. Comparisons between DPR and structural techniques often vary in methodology, making it imperative to systematically review the available data.

This review aims to critically evaluate the literature on functional outcomes after DPR, addressing: (1) patient-reported outcomes using validated instruments, (2) objective airflow measures and imaging-based nasal volume or valve dimensions, (3) comparative performance of DPR versus traditional methods and (4) the impact of DPR technique variations (e.g., “push-down” vs. “let-down”) on functional integrity.

By aggregating data from prospective cohorts, randomised controlled trials (RCTs) and a large case series, we aim to clarify whether DPR delivers on its promise of preserving, and often enhancing, nasal airway function.

## Materials and methods

### Search database

**Figure 1.**
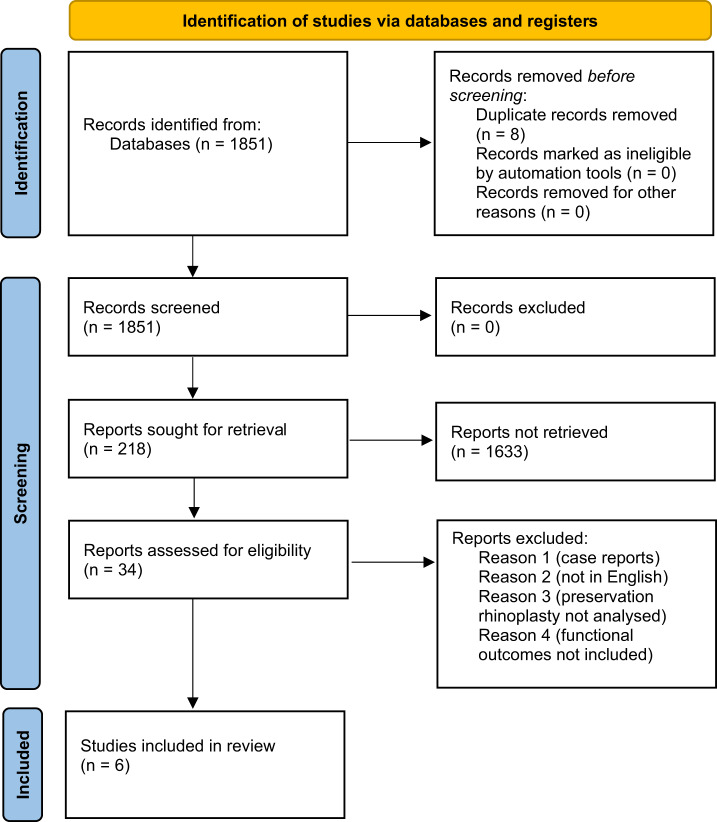
Identification of studies via databases and registers. Source: Page MJ, *et al. BMJ*. 2021;372:n71. doi: 10.1136/bmj.n71. This work is licensed under CC BY 4.0. To view a copy of this license, visit https://creativecommons.org/licenses/by/4.0/.

This systematic review was conducted using the Preferred Reporting Items for Systematic Review & Meta-Analyses (PRISMA) guidelines (Figure 1). A comprehensive literature search was performed across PubMed, Embase, Scopus and the Cochrane Library up to March 2025.

### Search terms

The following Medical Subject Headings terms and keywords were used: “dorsal preservation” AND “rhinoplasty”, “nasal obstruction” OR “nasal airway”, “functional outcome” OR “middle vault collapse”.

The following MeSH terms were used: rhinoplasty, dorsal preservation, nasal obstruction, functional outcome. The related-articles function was used to broaden the search. The reference list of retrieved papers and recent reviews were reviewed and included if relevant. Following the search, titles and abstracts were screened. Full text of potentially eligible articles were reviewed by two authors independently and eligible studies selected.

### Inclusion and exclusion criteria

#### Inclusion criteria

Inclusion criteria included the following: original studies (RCTs, cohort, case–control and case series ≥ 10 patients), DP rhinoplasty as primary procedure and post-operative assessment of nasal airway or obstruction.

#### Exclusion criteria

Exclusion criteria included the following: traditional hump resection rhinoplasty, isolated tip rhinoplasty and non-English articles, reviews and letters.

### Data extraction and quality assessment

Two independent reviewers extracted data on study design, patient demographics, surgical technique, assessment tools, incidence of nasal obstruction and interventions for obstruction. Quality assessment was performed using the Newcastle-Ottawa Scale for observational studies and the Cochrane Risk of Bias Tool for RCTs.

### Statistical analysis

Descriptive statistics were used on pooled patient cohorts.

## Results

### Study selection and characteristics

Following a comprehensive literature search and screening process, 218 unique records were identified. After removal of duplicates and screening of titles and abstracts, 34 full-text articles were assessed for eligibility. Of these, six studies met the inclusion criteria (Table 1). These included: 2 RCTs (Zarei *et al*.^12^ and Alsakka *et al*.^13^; three prospective cohort studies (Tas *et al*.,^14^ Emre *et al*.^15^ and Alan *et al*.^16^; and one large retrospective case series (Saban *et al*.^8^).

**Table 1.** Summary of included articles

Study	Design	Sample size	Comparison	Functional measures	Main findings
Tas and Erden 2021^14^	Prospective cohort	50	Let-down vs open with spreader graft	NOSE, SNOT−22, VAS	Both techniques improved function; no statistically significant difference between groups
Zarei *et al.* 2024^12^	Randomised controlled trial	84	DPR vs spreader flap	SCHNOS-O, VAS	No significant difference in functional or aesthetic outcomes
Emre *et al.* 2024^15^	Prospective cohort	124	DPR vs classical structural	Acoustic rhinometry, NOSE	No significant difference in airway dimensions or NOSE scores
Alsakka *et al.* 2024^13^	Randomised controlled trial	50	DPR vs dorsal hump reduction	SCHNOS, CBCT	Both groups showed functional improvement; no significant difference
Alan *et al.* 2023^16^	Prospective comparative study	34	Push-down DPR vs structural	Rhinomanometry, NOSE, SCHNOS	Both groups improved; higher nasal volume in DPR group
Saban *et al.* 2018^8^	Retrospective case series	320	None	Qualitative assessment	Excellent aesthetic and functional results; no dorsal irregularities or valve collapse

DPR = dorsal preservation rhinoplasty; NOSE = Nasal Obstruction Symptom Evaluation; SCHNOS = Standardized Cosmesis and Health Nasal Outcomes Survey; SNOT-22 = 22-item Sino-Nasal Outcome Test; VAS = Visual Analogue Scale

A total of 662 patients underwent dorsal preservation rhinoplasty. Sample sizes ranged from 34 to 320, with follow-up durations spanning from 6 months to over 24 months. All procedures were performed in tertiary rhinoplasty centres using various DPR techniques (push-down, let-down and superior strip).

### Patient-reported functional outcomes

Subjective outcomes were assessed using validated instruments including the NOSE score, SCHNOS - Obstruction subscale (SCHNOS-O) and VAS for breathing quality.

#### NOSE scores

NOSE scores decreased significantly post-operatively in all studies reporting it.^14^^–^^16^ DPR reduced NOSE scores by an average of 30–45 points, indicating marked improvement in perceived nasal airflow. No study found statistically significant differences in NOSE score improvement between DPR and conventional structural rhinoplasty techniques.

#### SCHNOS-O scores

Both RCTs reported significant post-operative reductions in SCHNOS-O scores (∼10–14 points) in both DPR and control groups.^12^^,^^13^ No statistically significant differences were noted between groups.

#### VAS scores for nasal obstruction

Where reported, VAS scores also improved significantly. Patients reported subjective improvement in nasal breathing after surgery, with reductions of 5–7 points on a 10-point scale.^16^ Overall, these findings suggest that DPR provides functional benefits that are comparable to traditional techniques, as reported directly by patients.

### Objective measures of nasal airway function

Objective functional outcomes were reported in three studies and assessed using acoustic rhinometry, rhinomanometry and cone-beam computed tomography (CBCT):

#### Acoustic rhinometry*^15^*

Post-operative assessments revealed increased minimum cross-sectional area (MCA) and nasal volume (VOL) after both DPR and structural rhinoplasty. The differences between techniques were not statistically significant, indicating that DPR does not compromise internal nasal geometry.

#### Rhinomanometry*^16^*

Both DPR and structural techniques led to reductions in nasal airflow resistance. While the DPR group showed slightly greater increases in nasal volume, these differences did not correspond with subjective differences in obstruction. Results confirmed functional equivalence between techniques.

#### CBCT volumetry*^13^*

Radiological analysis showed improvements in internal nasal valve angle and cross-sectional area in both DPR and conventional dorsal reduction groups. Again, no significant intergroup differences were observed. Collectively, these objective findings support the notion that DPR preserves or enhances nasal airway dimensions in the post-operative period.

### Post-operative nasal obstruction and complications

Post-operative nasal obstruction was rare and generally transient across all studies. When reported, it was most often attributable to: mucosal oedema in the early post-operative period, residual swelling at the internal nasal valve and hypertrophy of inferior turbinates. These symptoms were typically self-limited or responded to conservative measures such as intranasal corticosteroids. Only rare cases required additional intervention.

#### *Case series data* *^8^* *^,^* *^17^*

Among the 320 cases reviewed across both series, the overall revision rate was approximately 4 per cent. The incidence of nasal valve collapse or persistent obstruction was less than 2 per cent. These rates compare favourably with structural techniques and suggest that DPR maintains mid-vault and valve stability over time.

### Surgical technique variations

While most studies reported using push-down or let-down approaches, none found functional differences attributable to the specific DPR technique employed. The choice between DPR variants was based on anatomical indications such as dorsal height, septal alignment and keystone area configuration. All techniques preserved dorsal continuity and appeared equally effective in maintaining nasal function.

## Discussion

This systematic review demonstrates that DPR consistently improves nasal function. Post-operative NOSE, SCHNOS and VAS scores showed substantial reductions, often by 30 points or more in the case of NOSE, which reflects significant clinical benefit. These findings are comparable to those reported following traditional structural rhinoplasty, and importantly, no study reported inferior functional results with DPR.

These subjective improvements are supported by objective functional assessments. Studies employing acoustic rhinometry, rhinomanometry and CBCT volumetry confirmed that DPR maintains, and in some cases enhances, nasal airway patency.^13^^,^^15^^,^^16^ Measures such as minimum cross-sectional area and total nasal volume showed no detrimental changes post-operatively, with some studies reporting a modest increase in nasal volume in the DPR cohort.^13^^,^^15^^,^^16^ This may be attributable to the preservation of the mid-vault and keystone area, which helps maintain internal nasal valve patency and avoids iatrogenic narrowing, which can be seen with aggressive dorsal resection.

One of the core theoretical benefits of DPR lies in its ability to maintain the structural integrity of the nasal dorsum and mid-vault, avoiding the destabilisation that can be seen in structural techniques that require cartilage grafts or flaps to reconstruct the resected dorsum. The reviewed literature supports this advantage: mid-vault collapse, valve stenosis and related complications were rare, and revision rates were low.^8^^,^^17^ These findings reinforce the hypothesis that DPR not only simplifies operative technique by reducing the need for reconstruction but also offers functional protection through anatomic preservation.

The question of which DPR technique offers the most functional benefit remains open, as current evidence does not indicate any clear superiority among the various approaches. Push-down, let-down and cartilaginous push-down techniques all showed comparable outcomes in terms of both subjective symptoms and objective functional measures.^14^^,^^16^ The decision to employ one method over another appears to be guided more by anatomical considerations such as hump size, bony alignment and septal configuration than by clear evidence of functional advantage.

While this represents the first systematic review concentrating solely on functional outcomes after dorsal preservation rhinoplasty, several recent reviews have explored both cosmetic and functional aspects of DPR.^18^^–^^20^ These earlier works included broader aesthetic endpoints, whereas our review focuses specifically on functional patency and nasal airway preservation. Future studies integrating both dimensions would provide a more comprehensive understanding of DPR’s overall benefit.

There are some limitations to this review which should be discussed. The heterogeneity in surgical technique, outcome measures and follow-up duration precluded meta-analysis. Some studies relied solely on patient-reported outcomes without corroborating objective measures, and others lacked blinding or consistent application of inclusion criteria. Additionally, while follow-up in most studies extended to at least six months, there is limited evidence addressing very long-term (> five years) functional stability, which is particularly relevant for younger patients or those at risk of progressive structural changes.

Despite these limitations, the available evidence suggests that DPR is a functionally sound and aesthetically effective alternative to traditional techniques. It preserves native nasal architecture, minimises disruption to the nasal valve and mid-vault and yields functional outcomes that are at least equivalent, if not superior to traditional approaches.

**What is already known on the subject**

- Traditional dorsal hump reduction often requires mid-vault reconstruction (e.g., spreader grafts, flaps and sutures) to maintain function
- Structural disruption in classic techniques can risk mid-vault collapse, nasal valve compromise, irregular dorsal lines and airway obstruction
- Dorsal preservation rhinoplasty (DPR) maintains dorsal continuity, the keystone area and mid-vault stability, potentially reducing the need for grafting
- Advocates suggest DPR may offer superior functional outcomes by preserving natural anatomy, but concerns remain about possible airway narrowing
- Isolated reports and small series have described functional equivalence between DPR and structural rhinoplasty, but the evidence base has been fragmented

**What this paper adds to our understanding**

- Provides the first systematic review synthesising functional outcomes of DPR from randomised controlled trials (RCTs), cohort studies and large series
- Demonstrates consistent *improvement in patient-reported outcomes* (Nasal Obstruction Symptom Evaluation [NOSE], Standardized Cosmesis and Health Nasal Outcomes Survey [SCHNOS] and Visual Analogue Scale [VAS]) after DPR, equivalent to traditional structural methods
- Confirms through *objective measures* (acoustic rhinometry, rhinomanometry and CBCT volumetry) that DPR maintains or enhances nasal airway patency
- Shows *low complication and revision rates* (<5 per cent), with rare instances of persistent obstruction or valve collapse
- Establishes that functional outcomes are comparable across DPR technique variants (push-down, let-down), with no evidence of functional inferiority
- Supports DPR as a structurally and functionally safe alternative to traditional hump reduction rhinoplasty, reinforcing its role in modern rhinoplasty practice

## Conclusion

DPR reliably improves nasal airway function, while preserving structural integrity and requiring fewer reconstructive manoeuvres. This review supports DPR as a functionally sound, aesthetically viable approach for dorsal hump correction.
